# Prospective observational study of baloxavir marboxil in adults and adolescents with uncomplicated influenza from China

**DOI:** 10.3389/fmicb.2023.1292735

**Published:** 2023-11-27

**Authors:** Guichan Liao, Muye Xia, Yuanhui Jiang, Hongjie Chen, Wei Liao, Jie Peng, Shaohang Cai

**Affiliations:** ^1^Department of Infectious Diseases, Nanfang Hospital, Southern Medical University, Guangzhou, China; ^2^Intensive Care Unit, Sun Yat-sen University Cancer Center, Guangzhou, China

**Keywords:** influenza, baloxavir, oseltamivir, duration of fever, quality of life

## Abstract

**Introduction:**

There are limited data on the efficacy of baloxavir marboxil (baloxavir) versus oseltamivir in Chinese patients with influenza A.

**Methods:**

This study is an observational real-world investigation encompassing 246 patients (baloxavir, *n* = 147; oseltamivir, *n* = 99) confirmed positive for influenza A. The choice between baloxavir and oseltamivir antiviral treatments was determined collaboratively by the clinician and the patient. A thorough comparative analysis was undertaken between the two groups, examining parameters such as the duration of fever and symptoms, viral load dynamics, lymphocyte changes, and enhancements in health-related quality of life (QoL).

**Results:**

No significant differences were observed in demographic data between the two groups. The duration of fever was significantly shorter in the baloxavir group (*P* < 0.001). However, the duration of symptoms was not significant different (*P* = 0.167). Multivariable Cox analysis showed the independent factors affecting duration of fever were baloxavir treatment (HR = 2.033, *P* < 0.001), fever on day 1 (HR = 0.741, *P* = 0.010) and CRP level (HR = 1.009, *P* = 0.039). Moreover, sex (HR= 0.660, *P* = 0.019) and monocyte count (HR = 1.355, *P* = 0.018) were independent factors affecting the duration of symptoms. No significant difference in change of health-related quality of life (*P* > 0.05), positive rate of viral antigen on day 3 (*P* = 0.477) between the two groups. Remarkably, a mutation was observed in one case on the third-day after baloxavir treatment compared with first-day, from cysteine to serine at position 384 of the PA subunit.

**Conclusion:**

In the clinical setting, baloxavir demonstrated comparable clinical benefits to oseltamivir, establishing its efficacy as an effective antiviral therapy for Chinese patients with influenza.

## 1 Introduction

Influenza, a respiratory infectious disease caused by the influenza virus, is associated with severe complications, such as febrile seizures and influenza encephalopathy (Maleki et al., 2023). Currently, influenza continues to pose a significant global public health burden (Iuliano et al., 2018; Wang et al., 2020). Although influenza vaccines are effective, they require annual updates due to frequent antigenic drifts and occasional antigenic shifts (Sano et al., 2017; Kim et al., 2018). The global influenza surveillance and response system, established by the World Health Organization (WHO), predicts the strains for vaccine inclusion each year in advance (Uyeki et al., 2022). Notably, during the 2021–2022 period, China reported a modest 2.47% influenza vaccine coverage rate (Zhao et al., 2022), significantly lower than many countries, such as the United States (51.40%) (Grohskopf et al., 2022) and England (48.0%) (Oakley et al., 2021).

Antiviral medications play a crucial role in managing and treating influenza, particularly when vaccination is not administered or may not be fully effective. The Centers for Disease Control and Prevention (CDC) recommend a range of influenza treatments, including oral oseltamivir, inhaled zanamivir, intravenous peramivir, and oral baloxavir marboxil (baloxavir). The latter, serving as the prodrug of baloxavir, represents the latest addition to the category of anti-influenza medications. Baloxavir operates through an innovative mechanism by inhibiting the Cap-dependent endonuclease (CEN), a critical step in viral RNA transcription, thereby obstructing further viral replication (Yang, 2019). This novel mechanism has piqued the interest of the medical community and led to its approval in various countries. Initially sanctioned in Japan in February 2018, it subsequently received approval from the U.S. Food and Drug Administration (US-FDA) on 24 October 2018 (Heo, 2018). In China, baloxavir was granted an exemption from a phase III clinical trial and received approval for clinical application in 2021 for the treatment of influenza in patients older than 12 years of age.

The safety and efficacy of baloxavir in treating influenza were validated through a series of clinical trials, including one phase II trial and three phase III trials: CAPSTONE-1 (NCT02954354), CAPSTONE-2 (NCT02949011), and miniSTONE-2 (NCT03629184) (Hayden et al., 2018; Ison et al., 2020; Baker et al., 2023). The administration of baloxavir led to a significant reduction in viral load by approximately 2–3 logs compared to neuraminidase inhibitors (Noshi et al., 2018; Liu et al., 2022). In a double-blind, randomized phase III trial involving outpatients with influenza A infection, baloxavir demonstrated superiority over a placebo in alleviating influenza symptoms and outperformed both oseltamivir and the placebo in reducing viral load (Hayden et al., 2018; Noshi et al., 2018). A study has indicated that the simulated efficacy suggests Chinese patients could benefit similarly to their Asian counterparts (Liu et al., 2022). According to the WHO report, oseltamivir and baloxavir continue to be suitable options for the treatment of influenza virus infection. This recommendation is based on the fact that their substitutions associated with reduced susceptibility were infrequent, accounting for 0.6% (159 out of 26,010) for oseltamivir and 0.1% (18 out of 15,692) during the 2019–2020 periods (Govorkova et al., 2022).

However, despite its promising mechanism, real-world clinical data on the efficacy of baloxavir, especially in comparison to other established antiviral treatments like oseltamivir, remains limited, particularly in specific populations such as Chinese patients. In this study, we conduct an analysis of clinical data from Chinese influenza patients treated with either baloxavir or oseltamivir. Our analysis encompasses various factors, including the duration of fever and symptoms, viral load dynamics, lymphocyte change, and improvements in the health-related quality of life (HRQoL). The outcomes of this study offer valuable insights into the efficacy of baloxavir in influenza treatment, providing essential clinical support for its practical utilization in real-world scenarios.

## 2 Materials and methods

### 2.1 Study design

This observation study aimed to explore the clinical and virological efficacy of a single oral dose of baloxavir compared with two oral doses of oseltamivir daily for 5 days in patients infected with influenza during the June to August 2022 influenza season in three different outpatient department, in Nanfang Hospital, Southern Medical University, Guangzhou, China.

This study was approved by the Institutional Review Board of Nanfang Hospital, Southern Medical University (ID: NFEC-2022-272). All procedures were performed in accordance with the ethical standards of the responsible committee on human experimentation and the Declaration of Helsinki. All enrolled patients received adequate information on the nature, purpose, and possible risks and benefits of the trial as well as alternative treatment options. Informed consent was obtained from all patients included in the study.

### 2.2 Patients enrollment

Patients infected with influenza A virus who visited in outpatient clinic during the enrollment period were continuously included in the study. The inclusion criteria consisted of the following: (1) Patients aged 12 years or older; (2) onset of influenza-like symptoms (including fever, sore throat, cough, sneezing, or general malaise) within 48 h of their visit to the outpatient clinic; (3) confirmation of influenza A positivity using a rapid diagnostic test kit (Clearview Exact Influenza A & B) and subsequent validation in the hospital laboratory by polymerase chain reaction (PCR). Patients were excluded with underlying conditions, including pregnant women, those weighing less than 40 kg, and those with illness resulting in hospitalization. The choice of antiviral drugs, whether it be baloxavir or oseltamivir, was made collaboratively by the clinician and the patients. Patients were instructed to take the prescribed medication immediately upon receipt. It is noteworthy that patients in the baloxavir group were primarily recruited from the fever clinic, while patients in the oseltamivir group were drawn from three distinct outpatient settings. The disparity in enrollment was attributed to the fact that predominant choice of baloxavir treatment by most patients.

### 2.3 Demographic data and laboratory tests

Demographic information of the patients, including age, sex, height, weight, influenza vaccination, fever, time to treatment from fever onset, time to treatment from symptoms onset, and antipyretic use, were recorded. The duration of fever and associated symptoms were determined by the patients’ self-report. Duration of fever was measured from the first clinic visit, which was also the start of antiviral treatment, to the time of the last recorded fever (≥37.5°C) (Saito et al., 2020). The duration of symptoms was counted from the date and time of the first visit to the last record of a score of 1 or higher for symptoms including cough, sore throat, headache, nasal congestion, muscle ache, and fatigue. Severity of symptoms were self-reported by the patients on the 4-point Influenza Symptom Severity Scale (0 = no symptoms, 1 = mild, 2 = moderate, 3 = severe).

Blood cell counts were analyzed using a Sysmex SE9000 automatic blood cell analyzer. Serum alanine aminotransferase (ALT), aspartate aminotransferase (AST), albumin (ALB), and other serum biochemical parameters were measured using an Olympus AU5400 automatic biochemical analyzer. CD4^+^, CD8^+^, CD19^+^, and CD56^+^ lymphocyte counts were determined by flow cytometry. In this study, viral RNA load is detected from nasopharyngeal swabs. Influenza viral RNA was detected by PCR, and viral load was expressed as log_10_ copies/ml. Viral load was defined as 2 log_10_ copies/ml when viral RNA was positive but below the lower limit of detection (100 copies/ml). Viral subtypes were determined by means of Sanger sequencing in a 10:1 sampling of patients according to the order in which they were enrolled. Blood samples were obtained at baseline, defined as the beginning of treatment, and on day 7. Additionally, nasopharyngeal swabs were collected at baseline and on day 3 following treatment initiation. All tests and evaluations were performed according to the manufacturer’s instructions at the Department of Clinical Laboratory of Nanfang Hospital.

### 2.4 Sequencing analysis of PA gene

Polymerase acidic (PA) gene sequencing was conducted to assess the occurrence of amino acid substitutions linked to reduced susceptibility to baloxavir. Viral RNA in paired baseline and third-day post-treatment nasopharyngeal swabs from baloxavir treated patients, in whom concentrations of virus RNA were higher than the lower limit of sequencing on the third day after treatment. Sanger sequencing of the PA gene was executed after a nested PCR. For this study, the established lower sequencing limit for influenza A type PA genes was set at 10,000 copies/ml.

### 2.5 Health-related quality of life evaluation

The general HRQoL was evaluated using EQ-5D-5L instrument, a generic assessment tool comprising five dimensions: mobility, self-care, usual activities, pain or discomfort, and anxiety or depression (Herdman et al., 2011; Luo et al., 2017). In each dimension, there are five response levels, covering a range from 1 to 5 points, corresponding to the absence of problems, slight problems, moderate problems, severe problems, and extreme problems. In addition to the descriptive system, a Visual Analog Scale (VAS) was employed to measure participants’ health on a scale from 0 to 100. Lower scores on this scale indicate poorer quality of life (QoL) outcomes. The EQ-5D-5L questionnaire was administered to all patients on day 1 and on day 7 to evaluate the dynamic changes in their QoL.

### 2.6 Statistical analysis

Data were analyzed using the Statistical Product Service Solutions 23.0 (SPSS 23.0, Chicago, IL, USA) and compared using the Student’s *t*-test or Chi-square test. Differences in the duration of fever and symptoms were analyzed by the Kaplan–Meier test and log-rank test, respectively. Associated independent risk factors for the duration of fever and symptoms were identified by univariate and multivariate Cox regression analyses.

The propensity score (PS) matching method was employed to mitigate the impact of confounding variables in clinical features when comparing patients treated with baloxavir and oseltamivir. The model included demographic factors such as sex, age, and BMI, as well as the composite symptom score at baseline, body temperature at the initial visit, time from symptom onset to treatment, antipyretic, pseudoephedrine, and traditional Chinese medicine use. A logistic regression model was used to calculate a PS for each patient based on these confounding factors. Subsequently, patients in both groups were matched using nearest neighbor matching, with a maximum difference of 0.05 in PSs. The balance between the groups post-PS matching was assessed by computing the standardized difference, with values below 10% indicating similar baseline characteristics.

In multivariate analysis, adjusted model 1 included sex, age, BMI, on-demand antipyretic use, influenza vaccination, type of antiviral drugs, time to treatment from symptom onset, and fever at first day. Adjusted model 2 includes complete blood cells count, liver and renal function, albumin, globulin, LDH, CK, CKMB, HBDH, and C-reactive protein (CRP) levels. Adjusted model 3 includes the variables of model 1 and model 2. A *P-*value < 0.05 was considered statistically significant.

## 3 Results

### 3.1 Patients and demographics

A total of 180 patients treated with baloxavir and 111 patients treated with oseltamivir were recruited in the study. The demographics and clinical characteristics of all patients are shown in Supplementary Tables 1, 2. The PS-matching method yielded 147 and 99 patients in baloxavir and oseltamivir group, respectively. Comparison of demographics between the two groups showed no significant differences in sex ratio, age, height, and weight (Table 1). No significant difference was observed in the on-demand adjuvant drugs between the two groups, neither antipyretic nor pseudoephedrine use. Traditional Chinese medicine was used in 44.9% and 46.5% of patients in the baloxavir and oseltamivir group (*P* > 0.05). The percent of patients with influenza vaccination was extremely low and was comparable between the two groups.

**TABLE 1:** Demographics and clinical characteristics in patients enrolled at first visit day.

Characteristic	All patients		
	Baloxavir group *n* = 147	Oseltamivir group *n* = 99	*P*-value
Sex			0.245^a^
Male	81 (55.1%)	47 (47.5%)	
Female	66 (44.9%)	52 (52.5%)	
Age (years)	26.95 ± 0.8.71	28.69 ± 9.66	0.142^b^
Height, cm	165.64 ± 7.18	164.94 ± 8.15	0.484^b^
Weight, kg	59.34 ± 11.93	58.15 ± 11.92	0.445^b^
BMI, kg/m^2^	21.51 ± 3.72	21.24 ± 3.18	0.561^b^
Vaccinated with influenza, *n* (%)	2 (1.4%)	3 (3.0%)	0.394^b^
Fever at first visit day (°C)	38.58 ± 0.61	38.73 ± 0.65	0.187^b^
Composite symptom score at baseline	7.25 ± 3.50	6.99 ± 3.36	0.496^b^
Time to treatment from symptom onset (hours)	23.75 ± 8.50	25.09 ± 9.36	0.258^b^
**Concomitant medication history**			
Antipyretic, *n* (%)	122 (83.0%)	84 (84.8%)	0.728^a^
Pseudoephedrine, *n* (%)	36 (24.5%)	28 (28.3%)	0.554^a^
Cephalosporin, *n* (%)	10 (6.8%)	4 (4.0%)	0.414^a^
Traditional Chinese medicine, *n* (%)	66 (44.9%)	46 (46.5%)	0.896^a^
Loratadine, *n* (%)	2 (1.4%)	2 (2.0%)	1.000^a^
**Past medical history**			
Allergic rhinitis, *n* (%)	9 (6.1%)	4 (4.0%)	0.570^a^
Urticaria, *n* (%)	3 (2.0%)	2 (1.8%)	1.000^a^
Chronic kidney disease, *n* (%)	2 (1.4%)	2 (2.0%)	1.000^a^
Chronic bronchitis, *n* (%)	2 (1.4%)	1 (1.0%)	1.000^a^
Other disease^c^, *n* (%)	7 (4.8%)	2 (2.0%)	0.220^a^

The results of rapid screening showed that all patients were infected with influenza A virus, and the virus subtype was H3N2.

BMI, body-mass index.

^a^Chi-square test.

^b^Independent Student’s *t*-test.

^c^In the Baloxavir group, the recorded cases included, two cases of Favism, one case of Thalassemia, one case of Vitiligo, two cases of menstrual irregularity, and one case of chronic hepatitis B infection. Additionally, one case of thyroid nodule was recorded in the Oseltamivir group.

### 3.2 Duration of fever and influenza-related symptoms

**FIGURE 1:**
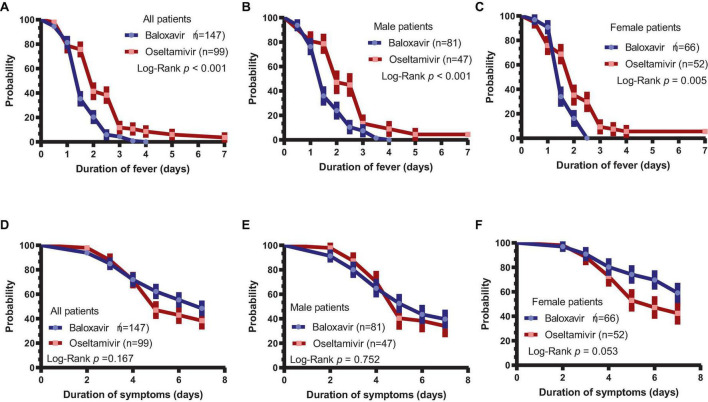
Duration of fever and influenza-related symptoms. **(A)** Duration of fever in the baloxavir group and oseltamivir group (*P* < 0.001). **(B)** Duration of fever in male patients between the baloxavir and oseltamivir groups (*P* < 0.001). **(C)** Duration of fever in female patients between the baloxavir and oseltamivir groups (*P* = 0.005). **(D)** Duration of symptoms in both treatment groups (*P* = 0.167). **(E)** Duration of symptoms in male patients between the baloxavir and oseltamivir groups (*P* = 0.752). **(F)** Duration of symptoms in female patients between the baloxavir and oseltamivir groups (*P* = 0.053).

The duration of fever was assessed in baloxavir- or oseltamivir-treated patients, and it was found that the duration of fever was significantly shorter in the baloxavir group [1.5 (1.0–2.5) days] than in the oseltamivir group [2.5 (1.5–3.0) days, *P* < 0.001], as supported by the Kaplan–Meier curve (Figure 1A, *P* < 0.001). In a subsequent subgroup analysis of both male and female patients, we observed that the duration of fever remained significantly shorter in the baloxavir group compared to the oseltamivir group, as illustrated in Figures 1B, C. However, the duration of influenza-related symptoms was not different between the two treatment groups (Figure 1D, *P* = 0.167). Furthermore, the duration of influenza-related symptoms was not significantly different between the two treatment groups in male subgroup (Figure 1E, *P* = 0.752), neither in female subgroup (Figure 1F, *P* = 0.053). Supplementary Figure 1 illustrates changes in each symptom during the course of treatment for both patient groups. Notably, the proportion of patients who had cough and throat pain were significantly lower in baloxavir group during the follow up (Supplementary Figures 1A, B, all *P* < 0.05). However, there were no statistically significant differences in change of other symptoms between the baloxavir group and the oseltamivir group (Supplementary Figures 1C–G, all *P* > 0.05), except fatigue on day 6.

### 3.3 Independent factors associated with fever and symptom duration

We conducted multivariable Cox analysis to evaluate independent factors associated with fever and symptom duration in the enrolled patients. As shown in Supplementary Table 3, when we included demographic data, multivariate analysis suggested that fever on visit day 1 was an independent factor affecting fever duration in enrolled patients [hazard ratio (HR) = 0.741, 95% CI 0.590–0.930, *P* = 0.010], except for baloxavir treatment (HR = 1.886, 95% CI 1.326–2.683, *P* < 0.001). When we included biomarkers from laboratory tests, the results showed that CRP was an independent factor (HR = 1.010, 95% CI 1.000–1.022, *P* = 0.034), as well as Monocyte (HR = 0.098, 95% CI 00.011–0.844, *P* = 0.035). When we included all markers, the results showed that baloxavir treatment (HR = 2.033, 95% CI 11.481–2.790, *P* < 0.001), fever on visit day 1 (HR = 0.741, 95% CI 0.590–0.930, *P* = 0.010) and CRP level (HR = 1.009, 95% CI 1.001–1.017, *P* = 0.039) were the independent factors affecting fever duration in the enrolled patients. Patients with baloxavir treatment, a higher CRP level and lower temperature at baseline had a shorter duration of fever.

Similar results were observed when we analyzed independent factors associated with symptom duration (Supplementary Table 4). Multivariate analysis showed that sex (HR = 0.671, 95% CI 0.477–0.946, *P* = 0.023) was an independent factor for symptom duration when only demographic data were included. When only laboratory markers were included, monocyte count was identified as an independent factor (HR = 1.440, 95% CI 1.118–3.569, *P* = 0.019). When all markers were included, sex (HR = 0.660, 95% CI 0.467–0.933, *P* = 0.019) and monocyte count (HR = 1.355, 95% CI 1.106–2.678, *P* = 0.018) were independent factors affecting the duration of symptoms in all enrolled patients. Female patients and patients with lower baseline monocyte counts had longer duration of influenza-associated symptoms.

**FIGURE 2:**
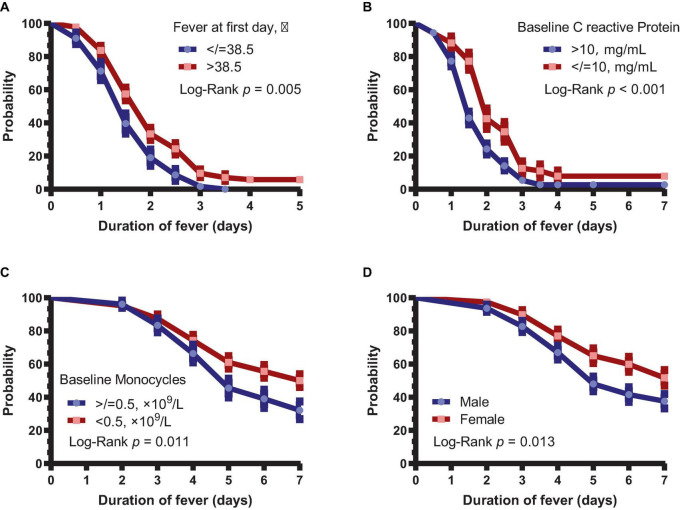
Subgroup analysis of the duration of fever and symptoms. **(A)** Duration of fever in patients with body temperature >38.5 and ≤38.5°C. **(B)** Duration of fever in patients with >10 and ≤10 mg/L CRP. **(C)** Duration of symptoms in patients with baseline monocytes <0.5 × 10^9^ and ≥ 0.5 × 10^9^/L. **(D)** Duration of symptoms between male and female patients.

Next, we analyzed the difference in fever duration between patients with body temperature >38.5°C and other patients on day 1. It was found that patients with a fever >38.5°C on day 1 had significantly longer fever than other patients (*P* = 0.005, Figure 2A). Similarly, duration of fever was shorter in patients with CRP >10 mg/L (*P* < 0.001, Figure 2B). Patients with baseline monocyte count of <0.5 × 10^9^/L had longer duration of symptoms (*P* = 0.010, Figure 2C). Moreover, the duration of symptoms was significantly longer in female patients than in male patients (*P* = 0.013, Figure 2D).

### 3.4 Changes in health-related quality of life in patient

**FIGURE 3:**
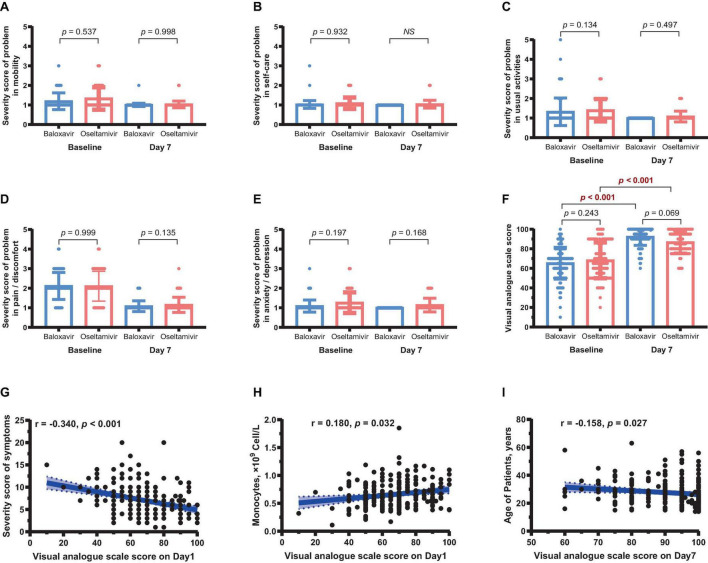
Changes in health-related quality of life in the two treatment groups. **(A–E)** Mobility, self-care, usual activities, pain/discomfort, and anxiety/depression scores of the two groups on day 7. **(F)** VAS score of both treatment groups. **(G)** Correlation between severity score of symptoms and VAS score (*r* = –0.340, *P* < 0.001). **(H)** Correlation between monocyte counts and VAS score (*r* = 0.180, *P* = 0.032). **(I)** Correlation between age and VAS score (*r* = 0.158, *P* = 0.027).

We also examined the changes in HRQoL at baseline and on day 7 of treatment in both groups. Although the QoL of all patients was increased in all five dimensions of mobility, self-care, usual activities, pain/discomfort, and anxiety/depression, there were no significant differences in the five dimensions of QoL between the two groups on day 7 (Figures 3A–E). The VAS score indicated that, despite the improved QoL in all patients, there was no significant difference in the self-assessed QoL score between the two groups (Figure 3F).

Significant negative correlation was found between the severity score of symptoms and the VAS score on visit day 1 (*r* = −0.340, *P* < 0.001, Figure 3G). Further analysis revealed that the VAS score was positively correlated monocyte counts (*r* = 0.180, *P* = 0.032, Figure 3H), but negatively correlated age (*r* = 0.158, *P* = 0.027, Figure 3I).

### 3.5 Changes in lymphocyte counts in patients

**FIGURE 4:**
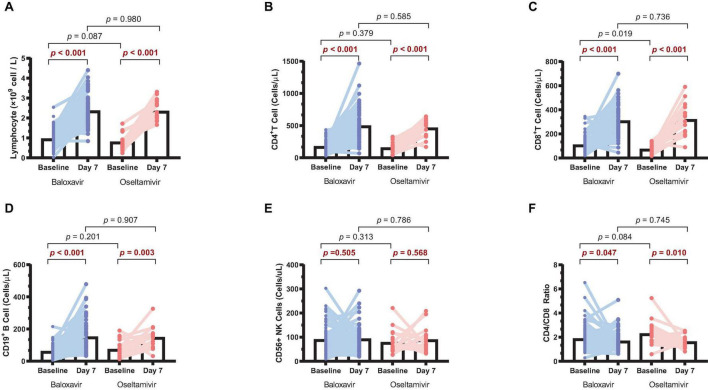
Changes in lymphocyte counts in the two treatment groups. **(A)** Lymphocyte counts on day 7 compared to baseline in both groups. **(B)** CD4^+^ T cell counts on day 7 compared to baseline in both groups. **(C)** CD8^+^ T cell counts on day 7 compared to baseline in both groups. **(D)** CD19^+^ B cell counts on day 7 compared to baseline in both groups. **(E)** CD56^+^ NK cell counts on day 7 compared to baseline in both groups. **(F)** CD4/CD8 ratio on day 7 compared to baseline in both groups.

Lymphopenia is a common clinical phenomenon in influenza patients. To conduct a more comprehensive analysis of the alterations in immune status among patients during antiviral treatments, we collected paired baseline and day 7 samples from 80 baloxavir-treated patients and 20 oseltamivir-treated patients. These samples were analyzed to assess changes in lymphocyte counts before and after treatment in both groups. It was found that lymphocytes on day 7 were significantly increased compared to baseline in both groups (Figure 4A). When we further analyzed lymphocyte types, it was observed that CD4^+^ T cells, CD8^+^ T cells, and CD19^+^ B cells were significantly increased in both groups (Figures 4B–D). Surprisingly, CD56^+^ NK cells were not increased after treatment in both groups (Figure 4E). Furthermore, there was a reduction in the CD4/CD8 ratio after treatment in both groups (Figure 4F). However, no statistically significant differences in lymphocyte counts or CD4/CD8 ratio were discerned between the two treatment groups on day 7.

### 3.6 Changes in virological status in patients treated with baloxavir

**FIGURE 5:**
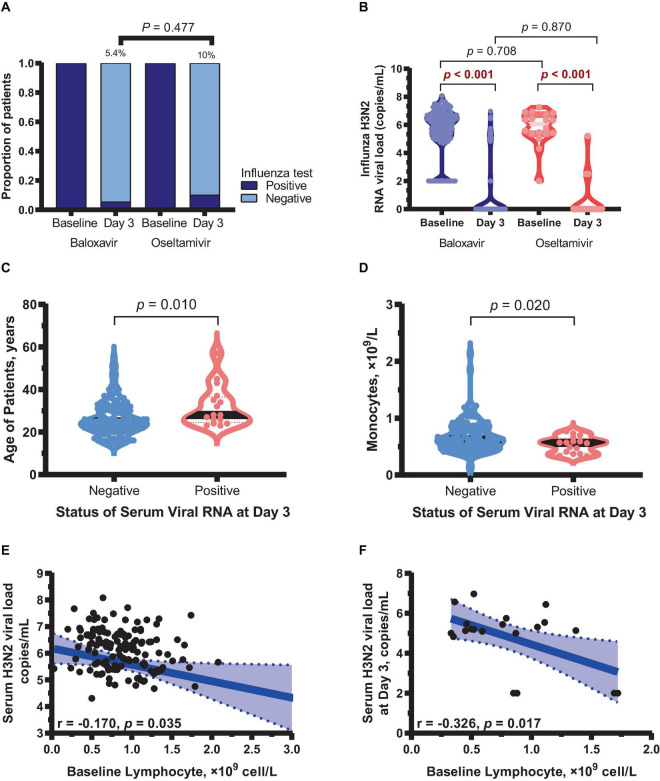
Changes in virological status in two treatment groups. **(A)** Influenza antigen rapid test results on day 3 in both groups. **(B)** Viral load on day 3 compared with day 1 in both groups. **(C)** Mean age of patients tested negative and positive for viral RNA on day 3. **(D)** Baseline monocyte counts in patients tested negative and positive for viral RNA on day 3 in both groups. **(E)** Correlation between lymphocyte counts and viral load at baseline. **(F)** Correlation between lymphocyte counts at baseline and viral load on day 3.

Rapid influenza virus antigen testing and H3N2 RNA viral load detection were performed on patients who had nasopharyngeal swabs on both day 1 and day 3 of disease onset. It was observed that 5.4% (8 of 147) patients in the baloxavir group were antigen positive on day 3, compared with 10% (2 of 21) in the oseltamivir group. However, the difference was not statistically significant (*P* = 0.324, Figure 5A). Similar changes were observed in viral load. Although viral load was significantly decline in both groups on day 3 compared with day 1 (both *P* < 0.001, Figure 5B), there was no significant difference in throat swab viral load between the two groups on day 3 (*P* = 0.870, Figure 5B). Interestingly, it was found that patients with negative viral load on day 3 were significantly younger than those with positive viral load positive (*P* = 0.045, Figure 5C). In addition, patients with negative viral load on day 3 had significantly higher baseline monocyte counts (*P* = 0.023, Figure 5D). Correlation analysis showed that lymphocyte counts at baseline were negatively correlated with viral load at baseline (*r* = −0.170, *P* = 0.035) and on day 3 (*r* = −0.326, *P* = 0.017) (Figures 5E, F).

Multivariable analysis was conducted to evaluate independent factors associated with virological response on day 3 (Supplementary Table 5). Age was an independent factor associated with virological response on day 3 when only demographic data [odds ratio (OR) = 0.958, 95% CI 0.911–0.999, *P* = 0.049] were included. When only laboratory markers were included, monocyte count was identified as an independent factor (OR = 1.799, 95% CI 1.273–4.808, *P* = 0.001). When all markers were included, age (OR = 0.963, 95% CI 0.914–0.997, *P* = 0.016) and monocyte count (OR = 2.690, 95% CI 1.049–5.766, *P* = 0.036) were independent factors affecting the virological response on day 3 in all enrolled patients.

We further analyzed the relationship between monocyte count and viral load. Patients with positive viral load on day 3 still had lower monocyte counts on day 7 compared with other patients (*P* = 0.016). The viral load on day 3 was negatively correlated with monocyte counts on day 1 (*r* = −0.23, *P* = 0.003) and on day 7 (*r* = −0.23, *P* = 0.006) (Supplementary Figure 2). However, we did not find a significant correlation between lymphocyte subtypes, including CD4^+^ T, CD8^+^ T, CD19^+^ B, CD56^+^ NK cells, and viral load (data not shown).

### 3.7 Identification of potential PA subunit mutations after baloxavir treatment

**FIGURE 6:**
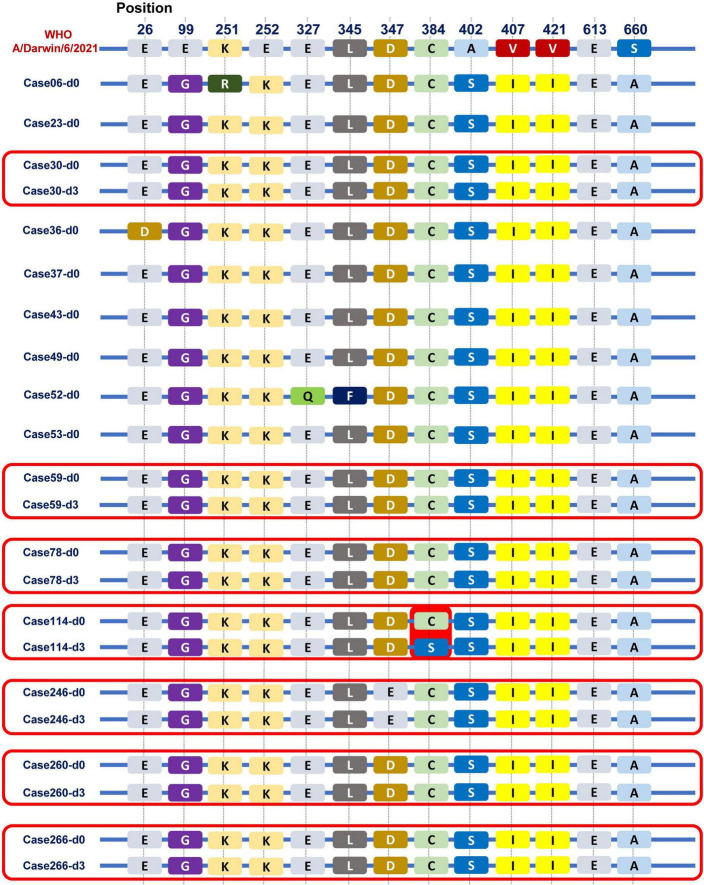
Viral PA subunit sequences before and after baloxavir treatment. We observed some differences in the PA subunit between the Guangzhou influenza virus strain in 2022 and the A/Darwin/6/2021 strain recommended by the WHO for the influenza vaccine. Especially, in one patient (1/7), we observed a mutation from cysteine to serine at position 384 (pa C384S) after baloxavir treatment.

Of total 180 patients treated with baloxavir, 15 patients (8.3%) had a positive detection of influenza viral load on day 3. We sequenced the paired first- and third-day of viral genomes and analyzed the amino acid composition of the PA subunit in these patients. Among them, nasopharyngeal swabs from eight patients on third day had low viral loads that were below the threshold for sequencing. Hence, we compared the PA subunit amino acid sequences on the first and third days for the remaining seven patients, as shown in Figure 6. Remarkably, in one patient (1/7), we observed a mutation from cysteine to serine at position 384 (paC384S) after baloxavir treatment. The PA subunit was referenced from the A/Darwin/6/2021 strain recommended by the WHO for the influenza vaccine.

## 4 Discussion

In our study, it was found that baloxavir can shorten the duration of fever more effectively than oseltamivir. Moreover, baloxavir can effectively shorten the duration of symptoms, reduce influenza viral load, and improve the QoL of Chinese patients with similar efficacy as oseltamivir. Although the two treatments have comparable effects on several endpoints, oseltamivir treatment is much longer (5 consecutive days) than baloxavir (single dose). Therefore, baloxavir may effectively avoids the problem of poor adherence in some patients while ensuring robust anti-influenza activity. It is worth noting that, to the best of our knowledge, this is the first efficacy study of baloxavir treatment for influenza conducted in China.

Clinical efficacy data on baloxavir in the Chinese population remain limited. Our study revealed that baloxavir-treated patients had shorter duration of fever, which was consistent with previously published result (Wagatsuma et al., 2022). Importantly, multivariate analysis identified baloxavir treatment as an independent factor affecting the duration of fever. Furthermore, it was found that fever on day 1 and CRP level were independent factors affecting the duration of fever. Similarly, we did not find a difference in duration of symptoms between the two treatment groups (Hayden et al., 2018; Wagatsuma et al., 2022). In the CAPSTONE-2 study, the median time to improvement of influenza symptoms was shorter in the baloxavir group than in the placebo group (*P* < 0.0001) (Ison et al., 2020). However, the median time to improvement of influenza symptoms was similar between the two treatment groups in our study. In addition, it was further found that monocyte count and sex were independent factors affecting the duration of symptoms. This study confirms that baloxavir is effective and safe in Chinese patients with influenza H3N2. However, this is an observational study focused on the treatment of H3N2 over a relatively short timeframe. Further evaluation of the clinical efficacy and safety of baloxavir is warranted for other influenza viruses or virus subtypes, including influenza B and H1N1.

In the present study, higher monocyte counts were found to be closely related to low viral load and shorter symptom cycle. Previous studies suggested that monocytes play an important role in influenza pathogenesis, especially in influenza A virus sensing and clearance (Shi et al., 2014; Duan et al., 2017). Type I interferon-induced lung monocyte recruitment is critical for influenza A virus clearance (Knapp et al., 2003). Moreover, a subset of lung monocytes can differentiate into moDCs to induce the accumulation of anti-influenza A virus cytotoxic T cells in the lung on day 7 post-infection, which suggests that influenza A virus clearance is reliant on cytotoxic T cells (Aldridge et al., 2009; Duan et al., 2017). Furthermore, Patel et al. (2021) found that a peripheral blood lymphocyte-to-monocyte ratio of <2 has diagnostic value in the acute phase of influenza and can be used for earlier detection and management of this disease and improving clinical outcomes. In our study, although after treatment, the lymphocyte-monocyte ratio in both groups have changed. However, we did not find antiviral treatment had an impact on the ratio. Moreover, we have also found no significant difference between two treatment groups at baseline and at day 7.

Lymphopenia is a prevalent clinical phenomenon among individuals with influenza. This phenomenon could be attributed to the retention of lymphocytes within the respiratory tract, including the pharynx, bronchi, and even lungs, following influenza infection. Consequently, this leads to a passive reduction in lymphocyte levels in peripheral blood. Our findings highlight that CD4^+^ T, CD8^+^ T, and CD19^+^ B cells, which play crucial roles in the immune response, may be the primary cell types affected by this lymphopenia. It is worth noting that we did not conduct functional studies on these specific cell types. Additionally, while we observed a negative correlation between the total lymphocyte counts and viral load, each subtype cell analyses did not reveal significant correlations with viral load. This may because immune process involved in viral clearance is a multifaceted outcome and is not contingent on a specific cell subtype. Moreover, the current study showed that all lymphocyte populations except CD56^+^ NK cells were increase after baloxavir or oseltamivir treatment. However, whether the increase in lymphocyte counts is also accompanied by restoration of cell functions remains unclear. Persistent decrease in lymphocyte function may trigger secondary infection (Damjanovic et al., 2012). Interestingly, it was also found that CD56^+^ NK cell count was still low on day 7. Previous studies have suggested that lung monocytes are involved in influenza A virus-induced secondary bacterial pneumonia (Jamieson et al., 2013; Duan et al., 2017). NK cells are also an important component in the control of influenza virus infection, which clear virus-infected cells and release antiviral cytokines (Jegaskanda et al., 2019). Therefore, dynamic observation of lymphocyte counts, assessment of lymphocyte functions, and monitoring of changes in monocytes and NK cells may help prevent secondary infection.

Indeed, it has been reported that influenza significantly contributes to a substantial decline in the QoL at the population level (Yang et al., 2017). Enhancing the QoL of influenza patients is an important treatment objective. Our study showed that the patients experienced a significant improvement in QoL after treatment with baloxavir or oseltamivir, indicating that both drugs can effectively improve the QoL of patients. Further analysis revealed that severity of symptoms was negatively correlated with VAS scores, while monocytes and albumin were positively correlated with VAS scores. In our study, we also found that ALB level was positively correlated with the VAS score. Previous study has suggested that low serum albumin levels resulting from inflammation-induced capillary leakage or disease-related anorexia during acute illness (Eckart et al., 2020).

It was previously shown that baloxavir can clear the virus more effectively and reduce infectious virus titers faster compared with oseltamivir (Noshi et al., 2018; Ison et al., 2020). However, the current study indicated that viral load was significantly decreased in both groups on day 3 but was comparable between the two groups. Specifically, only 5.4% of baloxavir-treated and 10% of oseltamivir-treated patients remained viral antigen positive on day 3 (48 h), which seems to be a shorter duration compared to previous study (a median of 96 h in oseltamivir) (Hayden et al., 2018). This may be attributed to not excluding the use of other medications in this real-world study, such as antipyretics, pseudoephedrine, cephalosporins, and traditional Chinese medicine. The impact of these medications on antiviral efficacy remains unknown. According to previous studies, ERK or MEK inhibitors can enhance the antiviral effect of baloxavir (Haasbach et al., 2013; Hamza et al., 2021). However, we did not find any statistically significant differences in clearance of virus between with or without using these medications, possibly the exist of confounding factors (such as dosage and frequency of use). Thus, further studies involved a rigorous design and a larger sample size are warranted. In addition, studies have suggested that baloxavir can reduce the possibility of household influenza transmission and be used as a post-exposure prophylaxis (Takashita et al., 2019; Ikematsu et al., 2020; Umemura et al., 2020). This effect could potentially be ascribed to the function of baloxavir to reduce the duration of virus clearance in patients. Nevertheless, further studies are required to confirm the post-exposure prophylactic effect of baloxavir within the Chinese population.

Influenza strains exhibited resistance in approximately 2.2 and 9.5% of patients who received baloxavir in the phase II and III trials, respectively (Hayden et al., 2018). A previous study showed that PA I38T/M emerged as the predominant baloxavir-resistant site, affecting up to 9.5% of cases (Yang, 2019). Moreover, substitutions at other PA residues (E23G/K, A37T, and E199G) were observed after baloxavir treatment (Ince et al., 2020; Jones et al., 2022a,b; Takashita et al., 2023). Given the high replication rate and lack of proofreading of the viruses under the selective pressure of antiviral treatment, the emergence of resistance to baloxavir was also considered in the current study. In this study, we noticed that one patient remained viral positive on the third day after baloxavir treatment, and a mutation was observed on the third day compared with baseline, from cysteine to serine at position 384 of the PA subunit (paC384S). Notably, other amino acid mutations have been documented, particularly in the context of H3N2, all localized within the amino acid sequence spanning positions 316 to 668 (Hayden et al., 2018). This finding suggests that this particular region may be predisposed to mutation when subjected to baloxavir treatment in the case of H3N2 influenza. Interestingly, the mutation identified at the PA 384 site in our study falls squarely within this critical interval. However, it is crucial to emphasize that this site has been identified with a mutation, and further experiments are necessary to confirm whether it possesses drug resistance functionality.

There are several limitations in this study. First, this is an observational clinical study and at a single center. Secondly, the number of patients enrolled was relatively limited due to the short period of the influenza epidemic. Second, it is important to note that our study had a limited follow-up duration, which raises questions about the long-term effectiveness of baloxavir in preventing complications. Finally, our study exclusively focused on outpatient populations, warranting further study to ascertain the treatment’s efficacy in inpatients with more severe symptoms. Nonetheless, it is essential to highlight the significance of this study within the current body of literature. There is a paucity of reports examining the clinical efficacy of baloxavir in the treatment of seasonal influenza, making this study a valuable addition to the existing knowledge.

## 5 Conclusion

In conclusion, it was confirmed that baloxavir is an effective treatment for Chinese influenza patients. The duration of fever in Chinese influenza patients is closely associated with fever on day 1 and CRP level, and symptom cycle is closely related to monocyte counts and sex. Baloxavir treatment significantly increased the total number of lymphocytes, decreased the viral load in throat swab samples, and improved the day 7 QoL of patients. However, a multicenter, prospective, real-world study with large sample size is required to confirm the efficacy of baloxavir in the Chinese population.

## Data availability statement

The original contributions presented in this study are included in this article/Supplementary material, further inquiries can be directed to the corresponding authors.

## Ethics statement

The studies involving humans were approved by the Institutional Review Board of Nanfang Hospital, Southern Medical University. The studies were conducted in accordance with the local legislation and institutional requirements. Written informed consent for participation in this study was provided by the participants’ legal guardians/next of kin.

## Author contributions

SC: Formal analysis, Funding acquisition, Investigation, Writing – original draft, Writing – review and editing. GL: Conceptualization, Data curation, Investigation, Methodology, Writing – review and editing. MX: Formal analysis, Investigation, Resources, Writing – review and editing. YJ: Methodology, Software, Writing – review and editing. HC: Methodology, Software, Supervision, Writing – review and editing. WL: Funding acquisition, Project administration, Validation, Visualization, Writing – review and editing. JP: Project administration, Validation, Visualization, Writing – review and editing.
