# Supplementary material for: Prospective observational study of baloxavir marboxil in adults and adolescents with uncomplicated influenza from China
Source: Front Microbiol. 2023 Nov 27;14:1292735. doi: 10.3389/fmicb.2023.1292735 (PMC10711061; doi:10.3389/fmicb.2023.1292735)
Supplement: Supplementary file 1 [file Data_Sheet_1.pdf]

Supplementary Table 1. Demographics and clinical characteristics in patients enrolled

Characteristic	All patients		
	Baloxavir group N=180	Oseltamivir group N=111	*P* value
**Sex**			0.072
**Male**	107 (59.4%)	54 (48.6%)	
**Female**	73 (40.6%)	57 (51.4%)	
**Age (years)**	27.19 ± 8.54	28.37 ± 9.42	0.272
**Height, cm**	166.14 ± 7.17	165.55 ± 8.15	0.595
**Weight, kg**	59.74 ± 11.73	58.35 ± 11.93	0.539
**BMI, kg/m^2**	21.53 ± 3.60	21.43± 3.78	0.540
**Fever at first day (℃)**	38.59 ± 0.63	38.69 ± 0.66	0.157
**Vaccinated with influenza**	2 (1.1%)	3 (2.7%)	0.310
**Composite symptom score at baseline**	7.22 ± 3.60	6.99 ± 3.38	0.558
**Time to treatment from symptom onset (hours)**	22.35 ± 8.77	25.25 ± 8.75	0.094
**Concomitant medication history**			
**Antipyretic**	154 (85.6%)	96 (86.5%)	0.864
**Pseudoephedrine**	46 (25.6%)	42 (37.8%)	0.035
**Traditional Chinese medicine**	79 (43.9%)	68 (61.3%)	0.005
**Cephalosporin**	30 (16.6%)	16 (14.4%)	0.741
**Loratadine**	2 (1.1%)	2 (1.8%)	0.637
**Past medical history**			
**Allergic rhinitis**	9 (5.0%)	6 (5.4%)	0.785
**Urticaria**	4 (2.2%)	2 (1.8%)	1.000
**Chronic kidney disease**	2 (1.1%)	4 (3.6%)	0.206
**Chronic bronchitis**	2 (1.1%)	1 (0.9%)	0.490
**Other disease**	7 (3.9%)	2 (1.8%)	0.490

Note: BMI, body-mass index.

Supplementary Table 2. Characteristics in patients in baloxavir group and oseltamivir group

Baseline Variable	H3N2 infected patients treated with		*P* value
	Baloxavir	Oseltamivir	
**WBC, $\times 10^9$/L**	7.06 ± 2.03	6.63 ± 1.76	0.355
**Neutrophils, $\times 10^9$/L**	5.37 ± 1.84	5.08 ± 1.79	0.492
**Eosinophils, $\times 10^9$/L**	0.21 ± 0.31	0.32 ± 0.41	0.154
**Monocytes, $\times 10^9$/L**	0.54 ± 0.37	0.44 ± 0.32	0.234
**Hemoglobin, g/L**	140.99 ± 15.49	140.62 ± 15.95	0.917
**RBC, $\times 10^{12}$/L**	4.95 ± 0.58	5.11 ± 0.65	0.222
**PLT, $\times 10^9$/L**	217.68 ± 53.59	235.33 ± 66.42	0.166
**PDW, fL**	11.69 ± 1.81	11.12 ± 1.65	0.171
**ALT, U/L**	22.17 ± 22.66	14.62 ± 11.91	0.135
**AST, U/L**	22.72 ± 14.01	20.05 ± 8.38	0.394
**Albumin, g/L**	48.86 ± 2.58	48.03 ± 3.36	0.180
**Globulin, g/L**	27.56 ± 3.26	27.62 ± 2.61	0.931
**TBIL, umol/L**	10.53 ± 5.78	10.08 ± 5.80	0.738
**DBIL, umol/L**	4.13 ± 1.85	3.94 ± 1.86	0.659
**Blood urea, mmol/L**	3.56 ± 1.06	3.73 ± 1.16	0.488
**Serum creatinine, umol/L**	86.93 ± 22.79	87.76 ± 24.97	0.876
**Blood uric acid, umol/L**	352.83 ± 96.59	333.24 ± 128.69	0.398
**eGFR, ml/min**	97.35 ± 17.39	100.21 ± 18.59	0.481
**LDH, U/L**	174.64 ± 33.84	167.52 ± 24.58	0.352
**CK, U/L**	118.67 ± 92.36	143.52 ± 119.97	0.262
**CK-MB, U/L**	12.51 ± 6.95	13.00 ± 5.22	0.753
**HBDH, U/L**	132.82 ± 23.53	128.95 ± 16.58	0.466
**CRP, mg/L**	15.77 ± 14.49	7.53 ± 6.36	0.011

Note: WBC, White blood cells; RBC, Eosinophils, Red blood cells, PLT, Platelets; PDW, Platelet distribution width; CRP, C reactive protein; ALT, Alanine aminotransferase; AST, aspartate aminotransferase; TBIL, total bilirubin; DBIL, direct bilirubin; LDH, lactate dehydrogenase; CK, creatine kinase; CK-MB, creatine kinase-MB isoform; HBDH, hydroxybutyrate dehydrogenase.

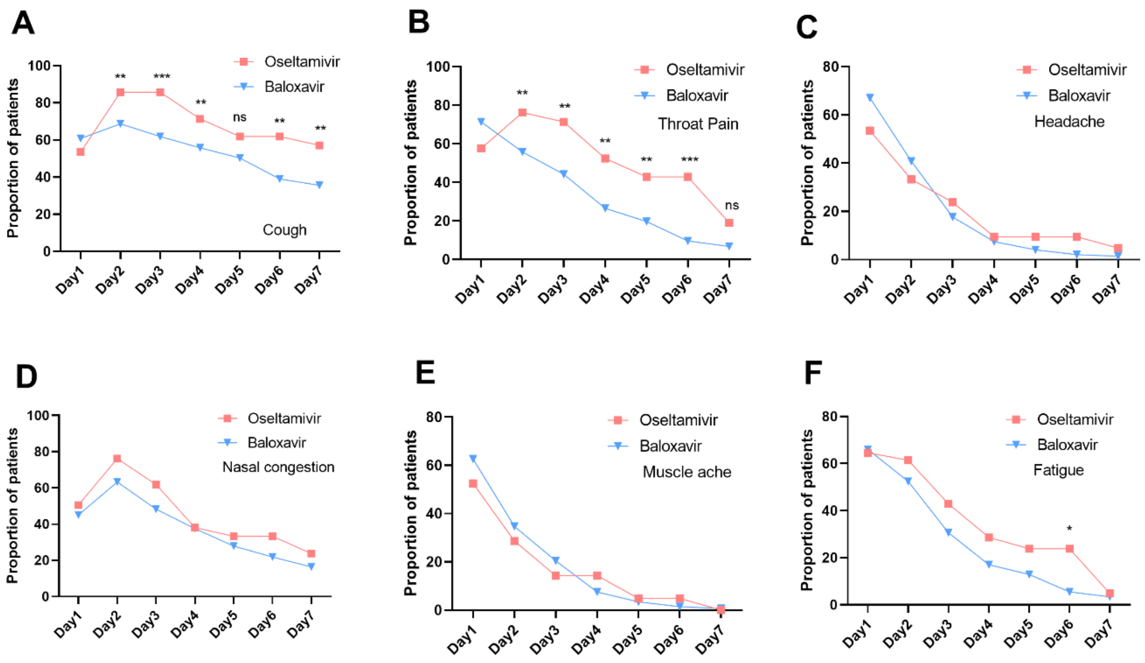

Supplementary figure 1 The duration of influenza -related symptoms.

A. The duration of cough in both treatment groups. B. The duration of sore throat in both treatment groups. C. The duration of headache in both treatment groups. D. The duration of nasal congestion in both treatment groups. E. The duration of muscle ache in both treatment groups. F. The duration of fatigue in both treatment groups. NS, no significant; * 0.05 < P < 0.01; ** 0.01 < P < 0.001; * ** P < 0.001.

Supplementary Table 3. Factors associated with duration of fever in patients (n=246)

	HR	95%CI	*P* value
**Adjusted Model 1**			
Fever at first day	0.688	0.552-0.857	0.001
Baloxavir use	1.886	1.326-2.683	<0.001
**Adjusted Model 2**			
C reactive protein	1.010	1.000-1.022	0.034
monocyte	1.098	1.011-1.844	0.035
**Adjusted Model 3**			
Baloxavir use	2.033	1.481-2.790	<0.001
Fever at first day	0.741	0.590-0.930	0.010
C reactive protein	1.009	1.001-1.017	0.039

Note: Adjusted model 1 included sex, age, BMI, on-demand antipyretic use, influenza vaccination, type of antiviral drugs, time to treatment from symptom onset and fever at first day. Adjusted model 2 includes complete blood cells count, liver and renal function, albumin, globulin, LDH, CK, CKMB, HBDH and CRP levels. Adjusted model 3 includes the variables of model 1 and model 2. A *P* value < 0.05 was considered statistically significant.

Supplementary Table 4. Factors associated with duration of symptoms in patients (n=246)

	HR	95%CI	*P* value
**Adjusted Model 1**			
Sex	0.671	0.477-0.946	0.23
**Adjusted Model 2**			
Monocytes, $\times 10^9$/L	1.440	1.118-3.569	0.019
**Adjusted Model 3**			
Sex	0.660	0.467-0.933	0.019
Monocytes, $\times 10^9$/L	1.355	1.106-2.687	0.018

Note: Adjusted model 1 included sex, age, BMI, on-demand antipyretic use, influenza vaccination, type of antiviral drugs, time to treatment from symptom onset and fever at first day. Adjusted model 2 includes complete blood cells count, liver and renal function, albumin, globulin, LDH, CK, CKMB, HBDH and CRP levels. Adjusted model 3 includes the variables of model 1 and model 2. A *P* value < 0.05 was considered statistically significant.

Supplementary Table 5. Factors associated with undetected viral load at day 3 (n=167)

	OR	95%CI	*P* value
**Adjusted Model 1**			
Age, years	0.958	0.911-0.999	0.049
**Adjusted Model 2**			
Monocytes, $\times10^9$/L	1.799	1.073-4.408	0.001
**Adjusted Model 3**			
Age, years	0.946	0.898-0.995	0.016
Monocytes, $\times10^9$/L	2.690	1.049-5.766	0.036

Note: Adjusted model 1 included sex, age, BMI, on-demand antipyretic use, influenza vaccination, type of antiviral drugs, time to treatment from symptom onset and fever at first day. Adjusted model 2 includes complete blood cells count, liver and renal function, albumin, globulin, LDH, CK, CKMB, HBDH and CRP levels. Adjusted model 3 includes the variables of model 1 and model 2. A *P* value < 0.05 was considered statistically significant. 147 patients treated with baloxavir and 20 patients treated with oseltamivir were included in this analysis.

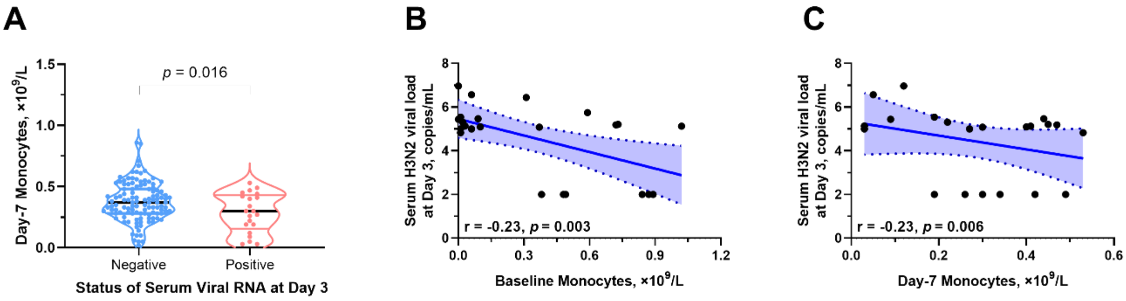

Supplementary figure 2 Relationship between monocytes and influenza viral load.

A. Monocyte counts on day 7 in patients tested negative and positive for viral RNA on day 3. B. Correlation between viral load on day 3 and baseline monocyte counts. C. Correlation between viral load on day 3 and monocyte counts on day 7. 147 patients treated with baloxavir and 20 patients treated with oseltamivir were included in this analysis.
